# Supplementary material for: Enzyme Bioprospection of Marine-Derived Actinobacteria from the Chilean Coast and New Insight in the Mechanism of Keratin Degradation in Streptomyces sp. G11C
Source: Mar Drugs. 2020 Oct 28;18(11):537. doi: 10.3390/md18110537 (PMC7693968; doi:10.3390/md18110537)
Supplement: Supplementary file 1 [file marinedrugs-18-00537-s001.zip › sup mat/marinedrugs-953443 supp mat figures pdf.pdf]

marine drugs

Article

Enzyme bioprospection of marine-derived Actinobacteria from the Chilean coast and new insight in the mechanism of keratin degradation in *Streptomyces* sp. G11C

Valentina González [1], María José Vargas-Straube[1], Walter O. Beys-da-Silva[2], Lucélia Santi[2], Pedro Valencia[3], Fabrizio Beltrametti[4] and Beatriz Cámara[1]*

Supplemental Figures

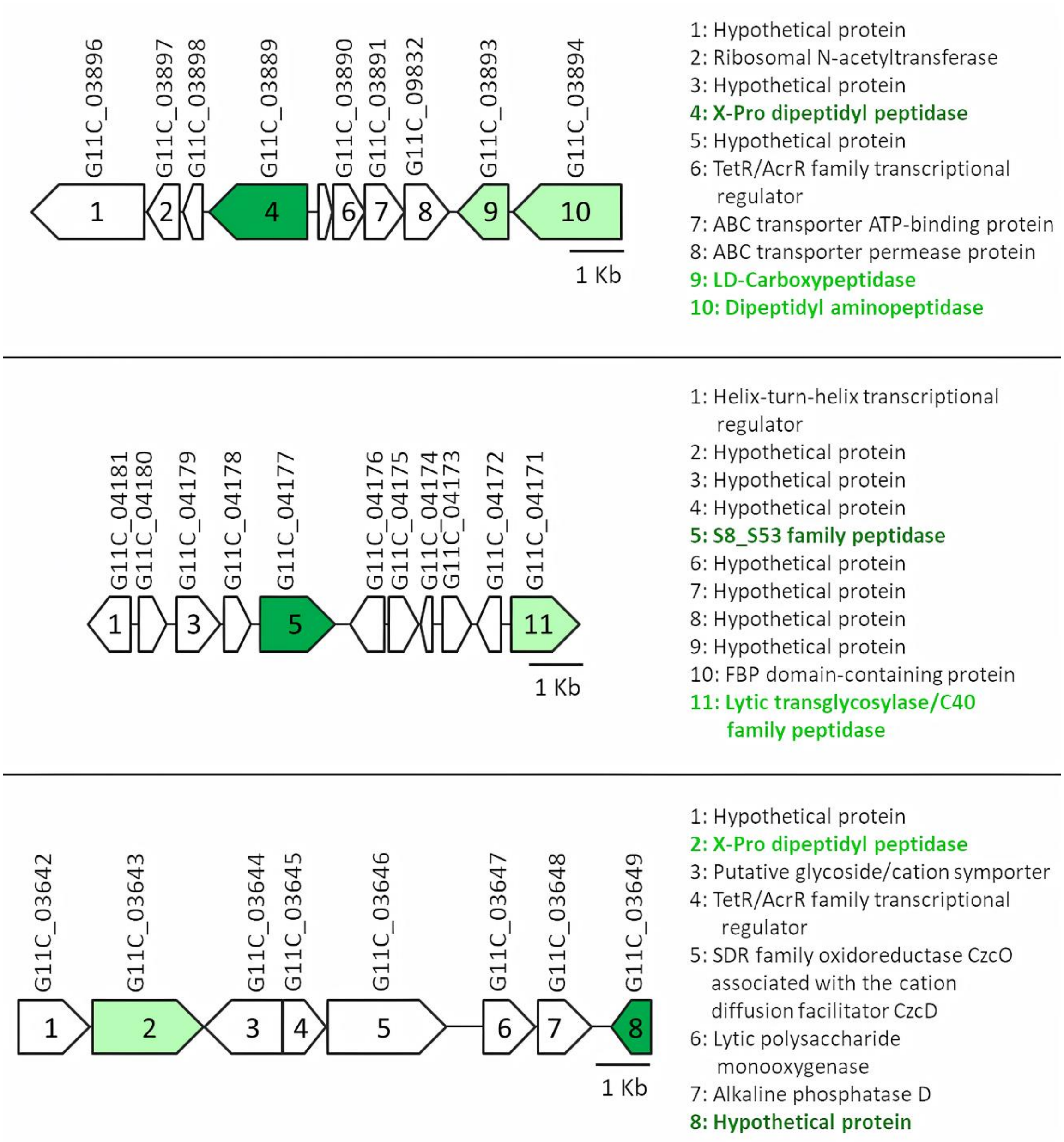

Figure S1. Genetic contexts of proteases identified in the secretome, close to other secreted proteases. The genetic contexts for the X-Pro dipeptidyl peptidase G11C_03889, the S8_S53 family peptidase G11C_04177 and the hypothetical protein G11C_03949 are shown.

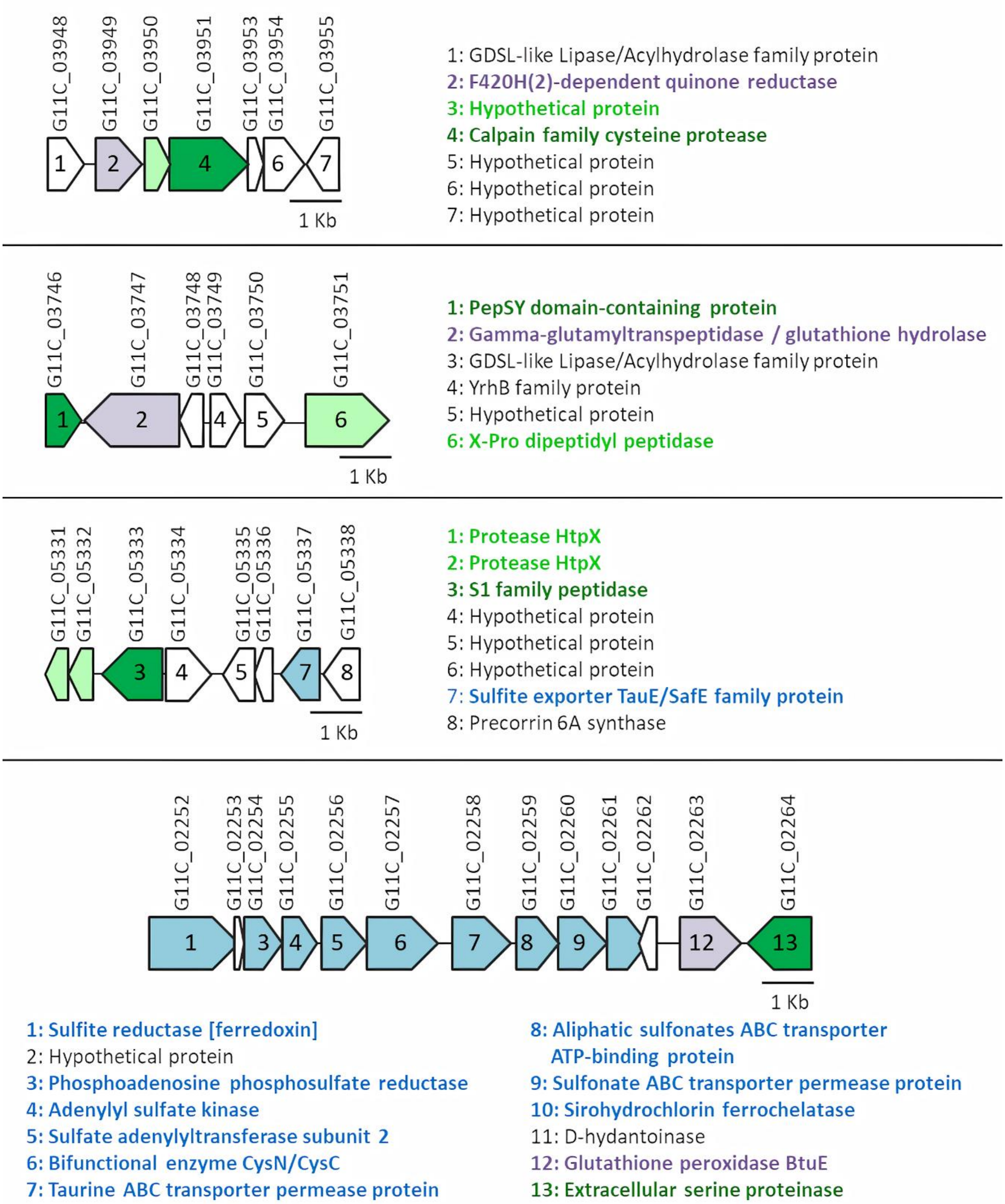

Figure S2. Genetic contexts of proteases identified in the secretome, close to genes encoding oxidoreductases and genes related to sulfite metabolism. The genetic contexts for the Calpain family cysteine protease G11C_03951, the PepSY domain-containing protein G11C_03746, the S1 family peptidase G11C_05333, and the extracellular serine proteinase G11C_02264 are shown.
